# A Case of Inoculated Cow-Pock Succeeded by a Confluent Eruption of Genuine Vaccine Vesicles on the Labia Pudendi; Occasioned by an Act of the Patient Herself

**Published:** 1804-05-01

**Authors:** 

**Affiliations:** Surgeon in Bridgnorth


THE
Medical and Phyfical Journal.

VOL. XI.] MAY 1, 1804. [NO. LXIII.

Printed for R. PHILLIPS, by W. Thorne, Red Lion Court, Fleet Street, London.

*A Case of inoculated Cow-Pock succeeded by a confluent Eruption of genuine Vaccine Vesicles on the* LABIA PUDENDI; *occasioned by an Act of the Patient herself. Communicated by* Mr. COLEY, *Surgeon in Bridgnorth.*

ON Feb. 29, 1804, I vaccinated three children of one family by inoculation. The vaccine virus was inserted into the skin of both the arms of each of them; for as they lived at a distance of nearly eight miles from my residence, I was desirous to effectually give them the disease with as little trouble to myself as possible. It so happened, that I had the virus (taken only a few days before) in a dried state, upon the points of two or three lancets, upon thread, and upon the convex side of a piece of quill. The six arms were vaccinated indiscriminately with the virus thus prepared; and each of the inoculations proved successful.

On the 8th of March I called upon these children, and observed the true vaccine vesicles formed, with a small surrounding inflammation or areola on each of the infected parts. The youngest child, a female, aged two years, which is the subject of this communication, had *scratched* the vesicles much; and I found, had been in the habit of so doing every night, and often in the day, except prevented by the woman who had the care of it. I directed measures to allay the increasing inflammation, and to prevent a repetition of this practice.

On the 12th I visited these patients, intentionally for the last time, when I found every thing had gone on well, except what I before noted respecting the habit the youngest child had acquired of scratching the inoculated parts, and which, though a good deal prevented, the nurse had not been able to interrupt; a slight fever had very perceptibly taken place in each of them; the vesicles had proceeded in the usual way, and a very extensive erythematous inflammation had occurred and was still present, but evidently declining. Conceiving they had perfectly undergone the vaccine disease, I now left them; previously however giving directions as to the surgical treatment of the inoculated parts.

On the 18th of March, being six days after the last time of my seeing these children, and eighteen days from the period of their being inoculated, I was desired, somewhat hastily, to go to examine the youngest of these subjects, who was represented to be very ill with a fever, retention of urine, a swelling of the labia pudendi, and a breaking out over all these parts. Surprised at such a representation of symptoms, I went immediately to see this child; and indeed, was not a little astonished at the appearances I had to observe, which were as follow: The labia pudendi were each of them swollen to a very great size, and exquisitely tender and inflamed; on the inside of each was a very considerably diffused pustular eruption of the *confluent* kind, many of the vesicles, as they doubtless were originally, having spread and run into each other, so as to form one continuous eruption, which was observable on separating the labia to go as far as the eye could perceive; on the fore parts of each labium were three or four very distinct and well formed *circular* pustules, evidently of the genuine vacciolous kind—so well marked, that no person who had ever been in the habit of seeing vaccine vesicles could possibly have mistaken them for any other; though this was not observed by the nurse, or the parents, or many others who had occasionally seen the child. The dysury, or pain of urining, was so distressing, that for many days they had not been able to prevail upon the child to pass its urine oftener than once a day. It had been costive, and there was a smart fever then present, and had been, as the attendants informed me, for some days.

Having thus sufficiently ascertained the state of the case, and being convinced in my own mind that the eruption was truly vacciolous, however it might have been occasioned; I thought it right to inquire with as much minuteness as possible into all the circumstances that were likely to throw any light on so important an anomaly of the disease: for if the system were occasionally, or under circumstances not previously discernible, liable to a secondary fever as the small-pox is, and moreover with that a confluent vacciolous eruption, such occasional occurrences, it is evident, would operate much against the new

 practice, practice, or tend ultimately to bring it into discredit. On inquiry how and when this disease of the pudenda was first discerned? the nurse very readily answered, that it had been discovered so long back as the time of my last visit, which was on the 12th (six days since); but as there was then only a little pustule or two, she did not think it worth mentioning; that what led to the discovery was her observing the child often rubbing and putting its fingers to those parts. When she first examined the pudenda, the child had then scratched so much as to make the part bleed. From the 12th to this period many fresh vesicles had formed; and lately she observed that they had run into one another, so as to make one continuous irregularly shaped eruption, occupying the whole of the inside of the labia, and proceeding inwards as far as she could see. The nurse and the parents expecting this eruption would have gone off, made no application for earlier assistance, nor did it appear that any thing had been locally used.

I at this time disguised my opinion of what the disease was; and prescribed such applications and medicine as the state of the case seemed to require.

Now, as to the cause or occasion of this secondary eruption and fever, I think it cannot fail being evident to any discerning practitioner, that the whole of these symptoms were brought on by a second, or perhaps many repeated inoculations of the parts mentioned, by the fingers of the child itself, who, it has been observed, through the whole progress of the disease, was constantly in the habit of scratching the inoculated parts of the arms; and with the nails thus infected with fresh vaccine fluid, and applied, as it evidently was, by scratching to such an extent as to produce an abrasion of the cuticle, and on parts too that are most numerously supplied with lymphatics, it cannot be wondered at that the consequence should be such as the symptoms described. I shall not determine so positively, whether the fever were strictly vacciolous, as I have not before seen any case of confluent vacciola; but as the fever, or febricula, that accompanies the inoculated cow-pock is uniformly of a very transitory duration, hardly indeed the fever of a day, it very probably was not truly a vacciolous fever, but a symptomatic fever arising from local irritation, inflammation, and pain of those parts.

On the 22d, the fever was entirely removed. The confluent pustules, from the applications of poultices and a saturnine lotion, were wholly separated, leaving the parts nearly healed and quite clean. The parts that had been

 occupied occupied by the distinct pustules were entirely healed; very superficial cicatrices remaining. In a few days after this I had the pleasure to leave the child in perfect health.

To this case, which in the present state of our knowledge of the Cow-pock I have thought right to lay before the public, I shall not subjoin any remarks; the facts stated are sufficiently clear, and the practical inference to be drawn from them is such as a medical practitioner cannot avoid noticing.

*Query.* Is it probable that some of the anomalous cases of the cow-pock, those accompanied with eruptions on parts not by the vaccinator inoculated, may have been occasioned by a similar cause?

*April,* 1804.

